# Enhancing Integrated Care Through the Voluntary and Community Sector: A Lean Management and Dynamic Capabilities Perspective

**DOI:** 10.5334/ijic.9014

**Published:** 2025-05-13

**Authors:** Huay Ling Tay

**Affiliations:** 1 School of Business, Singapore University of Social Sciences, Singapore

**Keywords:** integrated care, Voluntary and Community Sector, lean management, dynamic capabilities, efficiency, volunteer management

## Abstract

The Voluntary and Community Sector (VCS) is a critical contributor to integrated care, bridging gaps between health and social care services. However, operational inefficiencies and challenges in adapting to dynamic care environments hinder its full potential. This paper clarifies the perspective—drawn from research and practice—on applying Lean Management principles and Dynamic Capabilities Theory to enhance VCS operations. Using practical vignettes, it illustrates how these frameworks can improve volunteer recruitment, retention, and resource allocation, fostering efficiency and adaptability. Recommendations focus on specific aspects of integrated care, including service coordination, crisis adaptability, and volunteer management, leading to better patient outcomes.

## Context and Aim

Integrated care represents a coordinated, person-centred approach that bridges healthcare, social care, and community services to meet the increasingly complex needs of patients, particularly those with chronic conditions or complex social needs [1]. The Voluntary and Community Sector (VCS) plays a vital role in this system, offering services that complement the work of formal healthcare providers and ensuring that social determinants of health are adequately addressed [2]. The role of VCS organisations is critical in providing non-medical support, such as community outreach, health advocacy, and services that address isolation, mental health, and disability [3].

Despite their critical contributions, VCS organisations often face challenges sustaining their activities, optimising resource use, and scaling their services to meet growing demands within integrated care systems [4]. Key operational barriers include recruiting and retaining volunteers, resource constraints, and difficulties aligning their services with statutory care providers [5]. These challenges are compounded by the dynamic nature of healthcare systems, which necessitate adaptability and resilience from all sectors involved, including the VCS [6].

The aim of this paper is to provide actionable insights, based on research and practice, on how Lean Management [7] and Dynamic Capabilities [8] can address these challenges and enhance VCS contributions to integrated care.

## Practical Basis of the Perspective

The perspectives presented here are informed by applied research and practice-based insights from VCS organizations operating in integrated care environments. For instance, a workshop on Lean principles for volunteer management, conducted by the author for a group of VCS leaders, highlighted inefficiencies in recruitment pipelines and provided practical vignettes for improvements. Additionally, interviews with VCS leaders revealed the importance of adaptability during the COVID-19 pandemic that is similarly highlighted in the study by MacInnes et al, 2022 [17]. These real-world observations ground the theoretical frameworks in practical implementation.

## Volunteer Management, Recruitment and Retention

Volunteer management is a critical operational challenge for VCS organisations, with many struggling to recruit and retain volunteers whose skills match their needs [9]. Lean Management offers tools such as value stream mapping and root cause analysis to help streamline recruitment processes and better align volunteer skills with organisational goals [10]. For example, value stream mapping can help identify inefficiencies in the recruitment pipeline, ensuring that resources are focused on the most effective channels for attracting skilled volunteers. By eliminating wasteful processes, VCS organisations can improve the recruitment experience for both the organisation and the volunteer, increasing engagement and retention rates [10].

Once volunteers are onboarded, retaining them is crucial for maintaining the continuity of services within integrated care systems. The Lean Six Sigma DMAIC (Define, Measure, Analyse, Improve, Control) framework offers a structured approach to addressing volunteer turnover. By analysing the root causes of attrition—such as inadequate engagement or unclear role expectations—VCS organisations can implement targeted strategies to improve volunteer satisfaction and retention. Gazley (2013) found that applying Lean tools to volunteer management increased retention rates by 15% over two years in several large-scale VCS organisations, demonstrating the potential for these principles to drive sustainable improvements [11]. Schonberger, R. J. (2018) applied Lean Management principles to community healthcare organisations and found that process improvements led to a 20% reduction in administrative workload, allowing more time to be spent on patient-centred care activities [12].

## Operational Efficiency

Lean Management also offers practical solutions for improving operational efficiency in VCS organisations. By reducing waste and streamlining processes, Lean Management helps VCS organisations optimise the use of their limited resources [7]. For example, applying Lean principles to the administrative functions of VCS organisations can free up staff and volunteer time to focus on more value-added activities, such as direct service delivery or community outreach. Lean tools such as the SIPOC (Suppliers, Inputs, Process, Outputs, Customers) model can be used to map out critical processes and identify areas where efficiencies can be gained.

During the workshop, a VCS organization addressing food insecurity applied Lean principles by mapping recruitment workflows [18]. It identified bottlenecks, such as delays in application processing, and streamlined them through digital tools. This resulted in a reduction in onboarding time and higher volunteer satisfaction. To address retention, the same organization used root cause analysis to understand high volunteer turnover. Insights revealed that inadequate recognition and limited role clarity were key issues. Implementing a volunteer recognition program and clearer role descriptions increased retention.

## Organisational Adaptability

Dynamic Capabilities Theory emphasises the need for organisations to continuously adapt and evolve in response to changing environments [8]. In the context of integrated care, VCS organisations must be able to realign their resources and services to meet emerging demands, whether due to changes in government policy, funding availability, or the needs of service users. The COVID-19 pandemic provides a clear example of the importance of dynamic capabilities in the VCS sector. Many VCS organisations had to rapidly shift from in-person to remote service delivery models to continue providing essential services during lockdowns. Those with more robust dynamic capabilities—such as quickly deploying new technologies or reorganising volunteer roles—could better adapt to these changes and maintain service continuity [14].

Dynamic capabilities are particularly relevant in integrated care settings, where the needs of patients and communities are constantly evolving. For example, a VCS organisation focusing on mental health services might need to shift its focus towards addressing social isolation or supporting digital inclusion as new challenges arise in the integrated care system. By fostering a culture of continuous improvement and organisational learning, VCS organisations can enhance their ability to adapt to these changing circumstances, ensuring that they remain relevant and effective within integrated care [13].

## Enhancing Service Alignment with Integrated Care Goals

One of the key challenges VCS organisations face in integrated care is aligning their services with the broader goals of the healthcare system. Many VCS organisations operate independently of formal healthcare providers, leading to fragmented services that can undermine the effectiveness of integrated care. Dynamic Capabilities Theory provides a valuable framework for understanding how VCS organisations can better align their services with the needs of integrated care systems. This requires a focus on building strategic relationships with healthcare providers, engaging in continuous learning, and adapting services to meet the changing needs of patients and communities [15].

For example, a VCS organisation that provides mental health support might need to realign its services to meet the growing demand for telehealth services in response to changes in healthcare delivery models. By developing dynamic capabilities, such as the ability to integrate new technologies or reconfigure service delivery models rapidly, VCS organisations can ensure that they remain responsive to the evolving needs of integrated care systems. Valantiejienė et al. (2022) highlighted the importance of flexibility and adaptability in VCS organisations, noting that those with more robust dynamic capabilities were better able to collaborate with healthcare providers and align their services with integrated care goals [16]. During the COVID-19 pandemic, many VCS organisations had to rapidly redeploy their resources to meet the increased demand for digital services and remote support. Those with more robust dynamic capabilities were better able to make these adjustments, ensuring that they remained responsive to the needs of their communities [14].

## Conclusion and Recommendations

**Figure 1:**
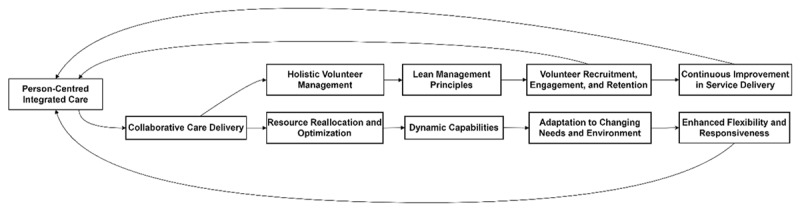
Conceptual Model Integrating Lean Management and Dynamic Capabilities for Person-Centred Integrated Care.

This perspective paper highlights the practical application of Lean Management and Dynamic Capabilities frameworks in enhancing VCS contributions to integrated care. Figure 1 shows a conceptual model to help visualise the role of the Voluntary and Community Sector (VCS) in Person-Centred Integrated Care through Lean Management and Dynamic Capabilities frameworks. The conceptual model illustrates the interconnection of key elements through Lean Management and Dynamic Capabilities. It shows how the VCS enhances integrated care by improving volunteer management, ensuring adaptability, and optimising resource allocation to maintain a person-centred focus.

Lean Management optimizes operations, while Dynamic Capabilities ensure strategic adaptability. Together, these frameworks enhance specific aspects of integrated care, such as improving service coordination, responding to crises, and optimizing volunteer management. By adopting these approaches, VCS organizations can deliver better outcomes, strengthen resilience, and sustain their role in integrated care systems.
